# Pediatric Critical Care Illness Severity Toolkit: Stata Commands for Calculation of Pediatric Index of Mortality and Pediatric Logistic Organ Dysfunction Scores

**DOI:** 10.2478/jccm-2023-0033

**Published:** 2024-01-30

**Authors:** Razvan Azamfirei, Colleen Mennie, James C. Fackler, Sapna R. Kudchadkar

**Affiliations:** Department of Anesthesiology and Critical Care Medicine, Johns Hopkins University School of Medicine, Baltimore, MD, USA; George Emil Palade University of Medicine, Pharmacy, Science, and Technology of Targu Mures, Romania; Department of Pediatrics, Charlotte R. Bloomberg Children’s Center, Johns Hopkins University School of Medicine, Baltimore, MD, USA; Department of Physical Medicine and Rehabilitation, Johns Hopkins University School of Medicine, Baltimore, MD, USA

**Keywords:** pediatric risk of mortality, PELOD, PIM3, STATA

## Abstract

**Introduction:**

Illness severity scoring tools, such as PRISM III/IV, PIM-3, and PELOD-2, are widely used in pediatric critical care research. However, their application is hindered by complex calculation processes, privacy concerns with third-party online calculators, and challenges in accurate implementation within statistical packages.

**Methods:**

We have developed a comprehensive, open-source toolkit for implementing the PIM-3, Simplified PIM-3, and PELOD-2 scores. The toolkit includes the pim3 and pelod2 commands and is compatible with Stata versions 12 and above. It features robust data validation, error messaging, a graphical interface, and support for SI and Imperial units. The toolkit's accuracy was validated through unit testing and synthetic data, comparing results with existing implementations.

**Results:**

In performance tests, the toolkit exhibited a median processing time of 21.82 seconds for PELOD-2, 14.06 seconds for PIM-3, and 9.74 seconds for Simplified PIM-3, when applied to datasets of 10,000,000 records. It consistently achieved 100% accuracy in both synthetic data tests and manual spot checks.

**Conclusion:**

The toolkit decreases processing time and improves accuracy in calculating pediatric critical care severity scores such as PELOD-2, PIM-3, and Simplified PIM-3. Its application in large datasets and validation highlights its utility as a tool for streamlining pediatric critical care research.

## Introduction

Outcome prognostication tools are indispensable in pediatric critical care research serving to control for the illness severity of pediatric intensive care unit patients. Several scores have been proposed, with the most notable being the Pediatric Risk of Mortality (PRISM III/IV), the Paediatric Index of Mortality 3 (PIM-3), and the Pediatric Logistic Organ Dysfunction-2 (PELOD-2) [1,2,3].

The application of each of these validated scoring systems involves complex, multi-step calculations. Online calculators are available for some of these scores, but they vary in their capacity to support multiple patients and the accuracy of their calculations. Additionally, submitting patient data to third-party websites may raise concerns about privacy and data protection. Implementing outcome prognostication scores in statistical packages is resource-intensive and prone to errors. As a result, researchers undertake custom implementation, leading to duplicated efforts and potential concerns about reproducibility if the implementation is incorrect.

Building on our previous work, where we detailed the development and implementation of a Stata command for calculating PRISM III and IV scores [4], this paper introduces a comprehensive toolkit for outcome prognostication in critically ill pediatric patients. This toolkit, including open-source implementations of the PIM-3, Simplified PIM-3, and PELOD-2 scores, aims to simplify these processes, thereby potentially reducing the challenges associated with their application in clinical research.

## Methods

We employed the software framework described in our previous work to develop the pim3 and pelod2 commands. The commands accurately implement the methods described in the original publications [1,2,3, 5]. Where necessary, each command is equipped with robust data validation, clear error messaging, a user-friendly graphical interface, and support for alternative units. This further reduces potential errors caused by inappropriate conversions. Clear and comprehensive documentation is provided for the usage of the commands. The commands are compatible with Stata versions 12 and above.

We have consolidated these commands into a toolkit for pediatric critical care research (*pccmtoolkit*). In addition to the *pim3* and *pelod2* commands, the toolkit includes the *prismscore* command as well as other utilities that streamline data management and processing. The entire toolkit, as well as the individual commands, are accessible on the Statistical Software Components (SSC) archive (https://ideas.repec.org/s/boc/bocode.html). Development versions of the toolkit are available on GitHub (https://github.com/razvanazamfirei/pccmtoolkit).

Each command was validated through unit testing, as well as synthetic data. The results were compared to other implementations of the score and manual spot checks were performed to ensure that calculations reflect the original methods. As described previously, we generated 10,000,000 patient records with random realistic data and predefined PELOD-2 and PIM-3 scores. Data generation was done through a separate process that did not rely on the developed command. Each command was benchmarked by performing calculations over 5 distinct sets of 10,000,000 patient records, using Stata/MP 18.0 2-cores. Results are presented as median (IQR). Institutional Review Board approval was not required.

## Results

The run-time results for each individual command are described in Table 1. The median time required to calculate scores for 10,000,000 records was 21.82 (21.40 – 22.31) seconds for PELOD-2, 14.06 (13.65 – 14.14) seconds for PIM-3, and 9.74 (9.71 – 9.98) seconds for the Simplified PIM-3 score. Each command achieved 100% accuracy in synthetic data tests, as well as in manual spot checks.

**Table 1.** Median run times for calculating pediatric severity scores.

**Score Type**	**Run 1** seconds	**Run 2** seconds	**Run 3** seconds	**Run 4** seconds	**Run 5** seconds	**Median (IQR) run time** seconds
PELOD-2	21.92	21.47	21.82	21.32	22.70	21.82 (21.40–22.31)
PIM-3	14.06	13.59	14.20	13.71	14.08	14.06 (13.65–14.14)
Simplified PIM-3	10.14	9.69	9.73	9.74	9.81	9.74 (9.71–9.98)

**Fig. 1.**
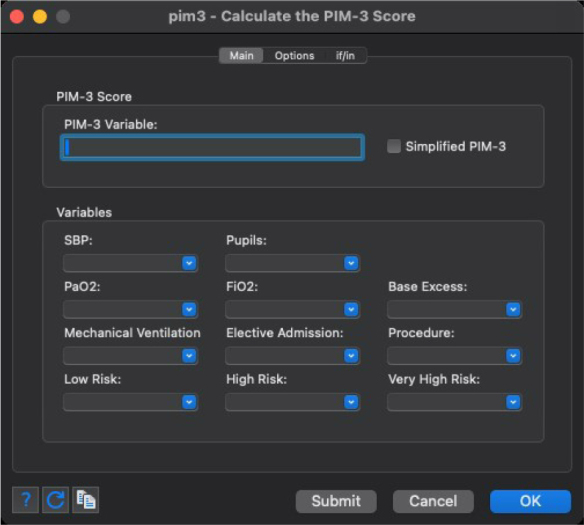
Stata dialog depicting the graphical user interface for the PIM-3 and simplified PIM-3 commands.

**Fig. 2.**
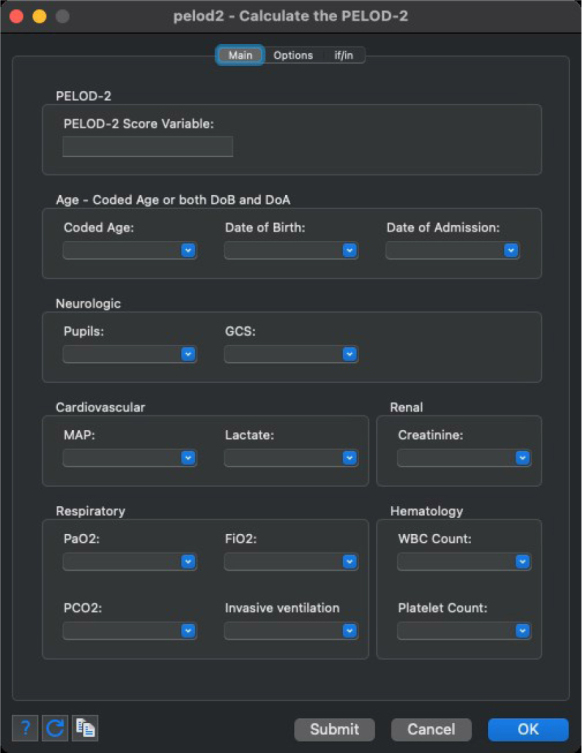
Stata dialog depicting the graphical user interface for the PELOD-2 command.

Additionally, the graphical user interface developed for these commands, which is depicted in Figures 1 and 2, enables intuitive variable specification and option selection. This interface streamlines the process for users, making it more accessible and efficient, particularly for those who may not be as familiar with traditional command-line operations.

## Discussion

This study demonstrates the robustness and efficiency of the developed commands for calculating PELOD-2 and PIM-3 scores, indicating that the commands are highly reliable for research purposes. The median run times for the calculations suggest that these commands can process large datasets within a feasible time frame. The development of this toolkit serves as a valuable resource for pediatric critical care researchers, now offering the potential to calculate multiple types of severity scores over the same dataset. However, it is important to note that the validity of the scores depends on the underlying quality of the data. Scores may use different definitions for comorbidities and require different intervals for using clinical data. The documentation provided with the commands describes some requirements and limitations; however, it is not a substitute for accurate data extraction, or appropriate data cleaning and management.

## Conclusion

Our work introduces a toolkit designed to enhance efficiency and accuracy in calculating the PELOD-2, PIM-3, and Simplified PIM-3 scores. Validation through synthetic data tests and manual spot checks confirmed 100% accuracy, ensuring reliability for research applications. This toolkit will serve as a resource for supporting pediatric critical care research, offering a streamlined approach to complex illness severity calculations.
